# Assessing the potential impact of artemisinin and partner drug resistance in sub-Saharan Africa

**DOI:** 10.1186/s12936-015-1075-7

**Published:** 2016-01-06

**Authors:** Hannah C. Slater, Jamie T. Griffin, Azra C. Ghani, Lucy C. Okell

**Affiliations:** Department of Infectious Disease Epidemiology, MRC Centre for Outbreak Analysis and Modelling, Imperial College London, London, W2 1PG UK

**Keywords:** Artemisinin resistance, Artemisinin combination therapy, Modelling

## Abstract

**Background:**

Artemisinin and partner drug resistant malaria parasites have emerged in Southeast Asia. If resistance were to emerge in Africa it could have a devastating impact on malaria-related morbidity and mortality. This study estimates the potential impact of artemisinin and partner drug resistance on disease burden in Africa if it were to emerge.

**Methods:**

Using data from Asia and Africa, five possible artemisinin and partner drug resistance scenarios are characterized. An individual-based malaria transmission model is used to estimate the impact of each resistance scenario on clinical incidence and parasite prevalence across Africa. Artemisinin resistance is characterized by slow parasite clearance and partner drug resistance is associated with late clinical failure or late parasitological failure.

**Results:**

Scenarios with high levels of recrudescent infections resulted in far greater increases in clinical incidence compared to scenarios with high levels of slow parasite clearance. Across Africa, it is estimated that artemisinin and partner drug resistance at levels similar to those observed in Oddar Meanchey province in Cambodia could result in an additional 78 million cases over a 5 year period, a 7 % increase in cases compared to a scenario with no resistance. A scenario with high levels of slow clearance but no recrudescence resulted in an additional 10 million additional cases over the same period.

**Conclusion:**

Artemisinin resistance is potentially a more pressing concern than partner drug resistance due to the lack of viable alternatives. However, it is predicted that a failing partner drug will result in greater increases in malaria cases and morbidity than would be observed from artemisinin resistance only.

**Electronic supplementary material:**

The online version of this article (doi:10.1186/s12936-015-1075-7) contains supplementary material, which is available to authorized users.

## Background

Artemisinin combination therapy (ACT) is the recommended first-line treatment for malaria in Asia and Africa. Artemisinin-resistant *Plasmodium falciparum* strains have emerged and spread within South-East Asia in recent years [1, 2], resulting in reduced treatment efficacy [3]. Concern has been raised about the potential impact on malaria morbidity and mortality if malaria parasites with similar levels of artemisinin resistance were to spread to or emerge independently in Africa, where 90 % of the global mortality from malaria occurs [1]. In the past, resistance to two major anti-malarials, chloroquine and sulfadoxine–pyrimethamine (SP), arose in South-East Asia and spread to Africa, indicating that the spread of artemisinin resistance is possible [4]. A molecular marker for artemisinin resistant *Plasmodium falciparum* malaria has been identified in Cambodia [5] and has subsequently been observed in Vietnam, Thailand and Myanmar [6]. Further analyses suggested that these artemisinin resistance associated mutations in the *P. falciparum* K-13 propeller gene have mainly resulted from independent emergences, rather than a geographical spread. Evidence of geographic spread between Cambodia and Vietnam exists, but there is no evidence of westward spread towards from Cambodia to Myanmar [6]. Similarly, there is evidence that K-13 mutations observed on the Thai-Cambodia and the Thai-Myanmar border have independent evolutionary origins [7].

Currently, few of the mutations associated with artemisinin resistance in South East Asia have been identified in Africa, and those few seen in Africa do not appear to be under selection [8, 9]. If artemisinin resistance were to develop in Africa, it is unknown whether it would spread from South East Asia or emerge independently. Modelling the spread of resistance within the continent is very difficult, as it could potentially emerge anywhere and in multiple locations. Therefore, in this study a simplified approach is adopted whereby the potential change in malaria burden is assessed at different fixed levels of artemisinin and partner resistance in each endemic country across Africa. Understanding the potential impact of resistance is essential for planning future control options, including the need for non-artemisinin alternatives for first-line therapy, and to quantify the costs and benefits of investing in resistance containment. A recent study estimated that widespread artemisinin resistance in Africa could result in US$385 million in productivity losses and US$32 million in additional medical costs per year [10].

Parasites can become resistant to either the artemisinin or the partner drug component of an ACT, or potentially, both. In the absence of resistance, the artemisinin derivative clears the bulk of the parasite biomass but has a very short half-life whereas the partner drug has a considerably longer half-life, meaning that it persists in the system long enough to clear any remaining parasites. Broadly speaking, artemisinin resistance is associated with slow parasite clearance (SPC) or early treatment failure (ETF), as well as causing recrudescence later after treatment [11, 12]. Artemisinin resistance is typically identified by monitoring the parasite clearance half-life [11] and proportion of individuals that are parasitaemic 3 days after treatment.

Partner drug resistance is more often associated with recrudescence around 3–4 weeks after treatment [12]. This results in re-emergence of the infection. Thus, identifying partner drug resistance involves following patients up for 28 or 42 days after treatment and regularly testing for parasites using PCR to determine whether the appearance of parasites is due to recrudescence or a new infection. Late clinical failure (LCF) is defined as clearing parasites by day 3, but having signs of severe malaria or parasitaemia with a temperature ≥37.5 °C between days 3 and 28 (or 42). Late parasitological failure (LPF) is defined as clearing parasites by day 3 but remaining parasitaemic between days 3 and 28 (or 42) and not having LCF.

Here the impact of these two types of resistance on malaria incidence and prevalence under scenarios of their development or spread in Africa is estimated by extending an existing malaria transmission model [13] to include the outcomes associated with artemisinin and partner drug resistance. Firstly fixed levels of resistance are considered under different transmission scenarios to explore the potential effects of artemisinin and partner drug resistance in terms of increased clinical incidence. Next, data from Cambodia and Africa are used to characterize five potential fixed resistance scenarios, which are used to estimate how different types of resistance could impact clinical incidence and prevalence across Africa. Separate resistant parasite populations are not explicitly modelled, nor are their spread over time and space, or any evolution in the degree of resistance, given the considerable uncertainty surrounding these issues.

## Methods

### Model description

**Fig. 1:**
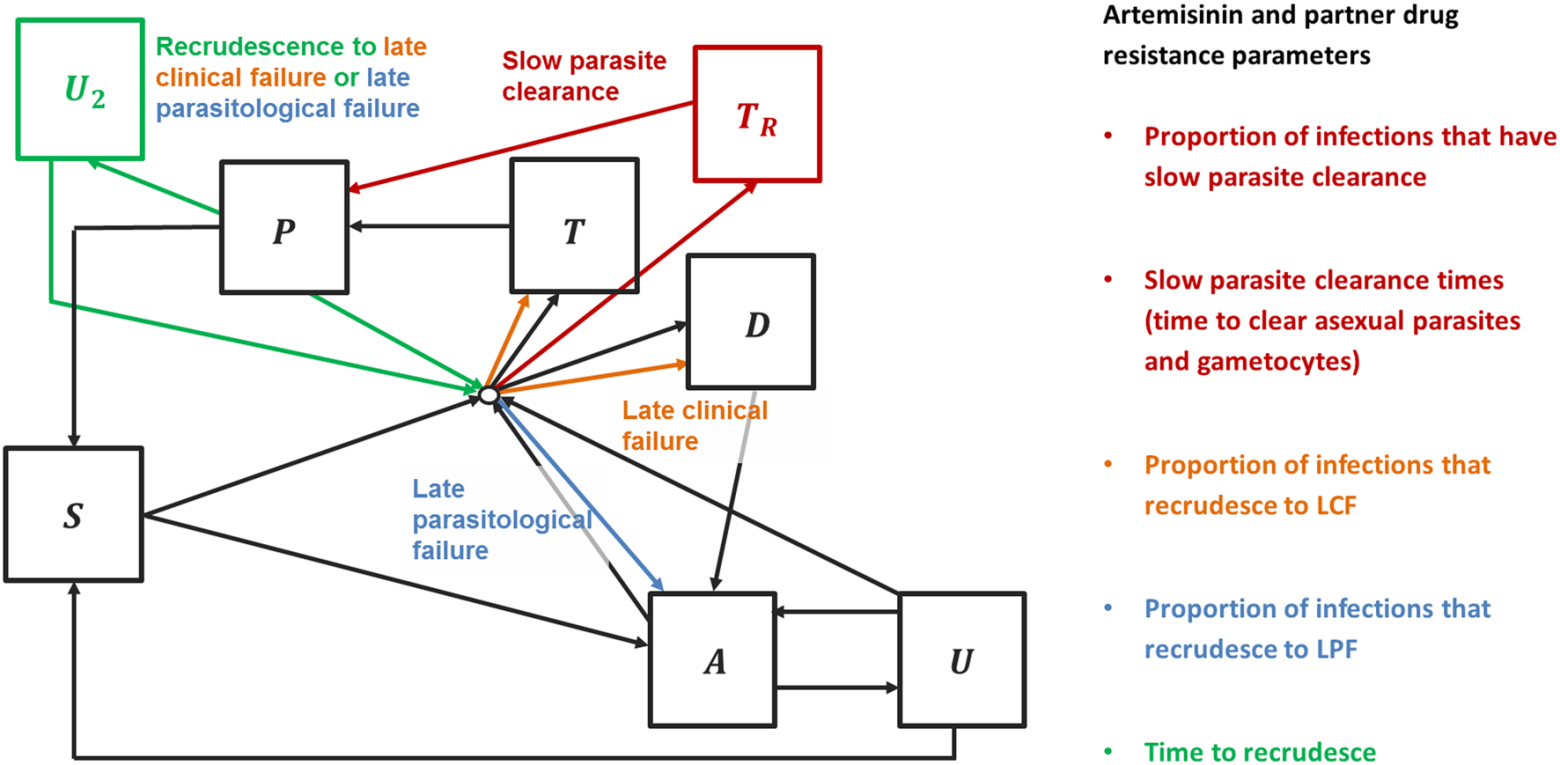
Malaria transmission model—human dynamics. Susceptible individuals (S) bitten by an infectious mosquito develop either asymptomatic infection (A) or clinical disease (T, T_R_, D) according to their level of immunity. Individuals treated for clinical disease with artemisinin-sensitive parasites (T) or artemisinin-resistant parasites (T_R_) clear infection (at different rates) and move to the prophylactically protected compartment (P) before returning to the susceptible compartment. Individuals with partner drug-resistant parasites can recrudesce from either the prophylactically protected compartment (P) or the post-treatment sub-patent infection compartment (U_2_) to clinical disease or asymptomatic infection. Prophylactically protected individuals can be re-infected, but with a lower probability than those in the fully susceptible state, based on the level of protection from the anti-malarial. Super infection can occur for asymptomatic patent (A) and sub-patent (U) infected individuals

A new model of drug resistance is developed within an existing age-structured individual-based mathematical model which describes the full transmission cycle of the parasite between humans and mosquitoes, as well as disease progression in humans. A brief description of the original transmission model is presented in Fig. 1 and full details are given elsewhere [14]. The model is extended to incorporate the following four ways in which drug resistance could impact on an individual’s treatment outcome (full details and model equations are presented in the Additional file 1):

1) *Slow parasite clearance* (*SPC*)—The proportion of the population that are parasitaemic on day 3 after treatment is used as an indicator of slow clearance. Individuals with SPC are assumed to remain in the treated compartment for longer but do eventually clear parasites successfully (or to sub-microscopic levels) and progress to the prophylactically protected compartment. This outcome is modelled by tracking an additional state T_R_ with an associated longer duration.

2) and 3) *Late clinical and parasitological failure* (*LCF and LPF*)—It is assumed that individuals with partner-drug-resistant parasites will recrudesce to either clinical (LCF) or asymptomatic (LPF) infection. A recrudescing individual initially clears parasites to a sub-microscopic level and enters the protected state (P) for a given period depending on the drug they have taken. They then move to a new state (U_2_) where they have sub-microscopic infection but are no longer protected. Individuals can recrudesce from the P or the U_2_ states to either clinical (LCF) or asymptomatic infection (LPF).

4) *Reinfection*—It is assumed that a protected individual (P) can become infected if challenged with resistant parasites. It is also assumed that an individual with partner-drug-resistant parasites can become re-infected before the existing infection recrudesces, in which case the new infection dominates.

The outcome of early treatment failure is not considered here—defined as having danger signs of severe malaria on days 1–3, having a higher parasite load on day 2 than day 0, having parasites on day 3 with a temperature ≥37.5 °C, or parasitaemia on day 3 being ≥25 % of the count on day 0—since this type of treatment failure is very rarely identified in individuals with artemisinin or partner drug resistant parasites. The outcome of severe malaria is also not considered, since it is unclear to what extent treatment can reduce the risk of an infection developing into a severe case, and how this would change with drug resistance.

An individual’s existing immunity to malaria will determine whether their recrudescing infection is either clinical or asymptomatic. It is assumed that a recrudescing individual in a high prevalence area has a higher probability of developing asymptomatic infection rather than clinical infection compared to an individual in a low prevalence area due to having greater acquired immunity. This is important when extrapolating from Cambodia to higher prevalence areas in Africa [15].

### Generic model simulations

Firstly, the impact of varying levels of LPF, LCF and SPC on clinical incidence in five transmission intensity settings (1, 5, 10, 25 and 50 % parasite prevalence in 2–10 year olds) is investigated to understand which of the three outcomes associated with artemisinin and partner drug resistance results in the greatest increase in incidence, and how this result is sensitive to the transmission intensity. In each simulation, it is assumed that a fixed proportion of parasites circulating in a population are resistant.

### Resistance scenarios and parameter values

Data were collated from three studies in Cambodia and two in Africa where varying levels of artemisinin and partner drug resistance were found (Table 1). These data were used to parameterize five potential resistance scenarios.

**Table 1:** Artemisinin and partner drug studies used to inform the resistance scenarios

Location	Pailin, Cambodia	Pursat, Cambodia	Oddar Meanchey Province, Cambodia	Tasanh, Cambodia	Sudan and Uganda
Reference	Leang et al. [16]	Leang et al. [16]	Spring et al. [17]	Bethell et al. [18]	Mukhtar et al. [19]^a^; Priotto et al. [20]^b^
Drugs given	DHA-PQP	DHA-PQP	DHA-PQP	AS	AS + SP
Percentage of treated individuals with slow parasite clearance	32.2 %	8.6 %	54 %	48.2 %	3.5^b^ %
Slow parasite clearance time (days)	8.91^c^	6.04^c^	10.26	10.86^c^	5.42^c^
Percentage of treated individuals that recrudesce to LCF	2.7 %	5.4 %	21 %	1.6^d^ %	3.9^a^ %
Percentage of individuals that recrudesce to LPF	10.7 %	0 %	24 %	4.8^d^ %	10.4^a^ %
Time to recrudesce (days)	26.9	25.6	27.1	24.5^e^	25.3^a^
Summary	Medium artemisinin and partner drug resistance	Low artemisinin and partner drug resistance	High artemisinin and partner drug resistance	High artemisinin resistance	No artemisinin resistance and high partner drug resistance

^a,b^Denotes which study each value was obtained (a = Mukhtar et al. [19], b = Priotto et al. [20])

^c^These values were not given in the studies and were derived from data—see Additional file 1: Supplementary methods for details

^d^Values not given in paper, only total proportion of people recrudescing (6.4 %)—this was split up to match the LCF:LPF ratio from the 2010 Tasanh study

^e^Not given in paper, assumed to be same as 2010 Tasanh study

*Scenario 1*: *Pailin*, *Cambodia*. Based on results of a dihydroartemisinin-piperaquine (DHA-PQP) trial conducted in 2008–2010 where there appeared to be resistance against both the artemisinin component and the partner drug piperaquine [16] as indicated by SPC and recrudescence rates of >10 %. The location represents an intermediate resistance scenario.

*Scenario 2*: *Pursat*, *Cambodia.* From the same study as scenario 1, day three positives and some recrudescence were observed after DHA-PQP treatment in Pursat, but at a much lower level than Pailin, although a higher proportion of recrudescing individuals developed clinical symptoms compared to scenario 1 [16]. This suggests some degree of artemisinin resistance but very low or absent piperaquine resistance. This location is used to represent a low resistance scenario.

*Scenario 3*: *Oddar Meanchey*, *Cambodia.* In this location day three positivity and recrudescence are very high, indicating high levels of resistance to artemisinin and the partner drug [17]. This is to date one of the highest observed treatment failure to an ACT anywhere in the world and represents the current worst-case scenario.

*Scenario 4*: *Tasanh*, *Cambodia.* The studies from this location used artesunate (AS) monotherapy for 7 days [18]. Recrudescence was found to be low, but day-3 positivity was high. This scenario is used to consider the impact of a parasite population resistant to artemisinin, but sensitive to the partner drug (with low recrudescence) based on the assumption that SPC would still have occurred in the presence of a partner drug.

*Scenario 5*: *Sudan and Uganda.* Data from two studies are combined to examine the efficacy of artesunate + sulfadoxine–pyrimethamine (AS + SP) [19, 20]. There is no confirmed artemisinin resistance in Africa to date, whereas SP resistance is widespread [4]. Therefore this example represents a scenario where the artemisinin component of the drug is fully effective but the partner drug is failing.

### Africa model simulations

The potential impact of artemisinin and/or partner drug resistance in Africa is estimated by simulating the impact of each of the five resistance scenarios. Simulations were run at the resolution of the first administrative unit across Africa (sub-national regions) [14]. At this spatial unit, four data layers were incorporated: (1) underlying population demographics [21, 22], (2) parasite prevalence in 2010 [23], (3) seasonal patterns of transmission [24], and (4) intervention coverage: including access to treatment and LLIN coverage [14, 25–27] (see Additional file 1 for details). For each resistance scenario the clinical incidence and parasite prevalence (as detected by microscopy or RDTs) are simulated for each first administrative unit in Africa from 2016 to 2020 and compare it to a scenario with no resistance. For simplicity it is assumed that all anti-malarials in use are artemisinin-based combinations which, although not currently the case, is likely to reflect future treatment trends. ACT coverage is defined as the proportion of symptomatic malaria cases receiving an anti-malarial. To simplify simulations, it is assumed that resistance to the artemisinin component or partner drug is uniform across Africa as characterized in the resistance scenarios, regardless of which ACT regimens are used in different parts of Africa. Country-specific yearly estimates of LLIN scale-up up to 2012 are incorporated and it is assumed that coverage remains at the 2012 levels in future years. Resistance is introduced in 2012, so by 2016, the model has reached a new equilibrium.

## Results

### Generic model simulations

**Fig. 2:**
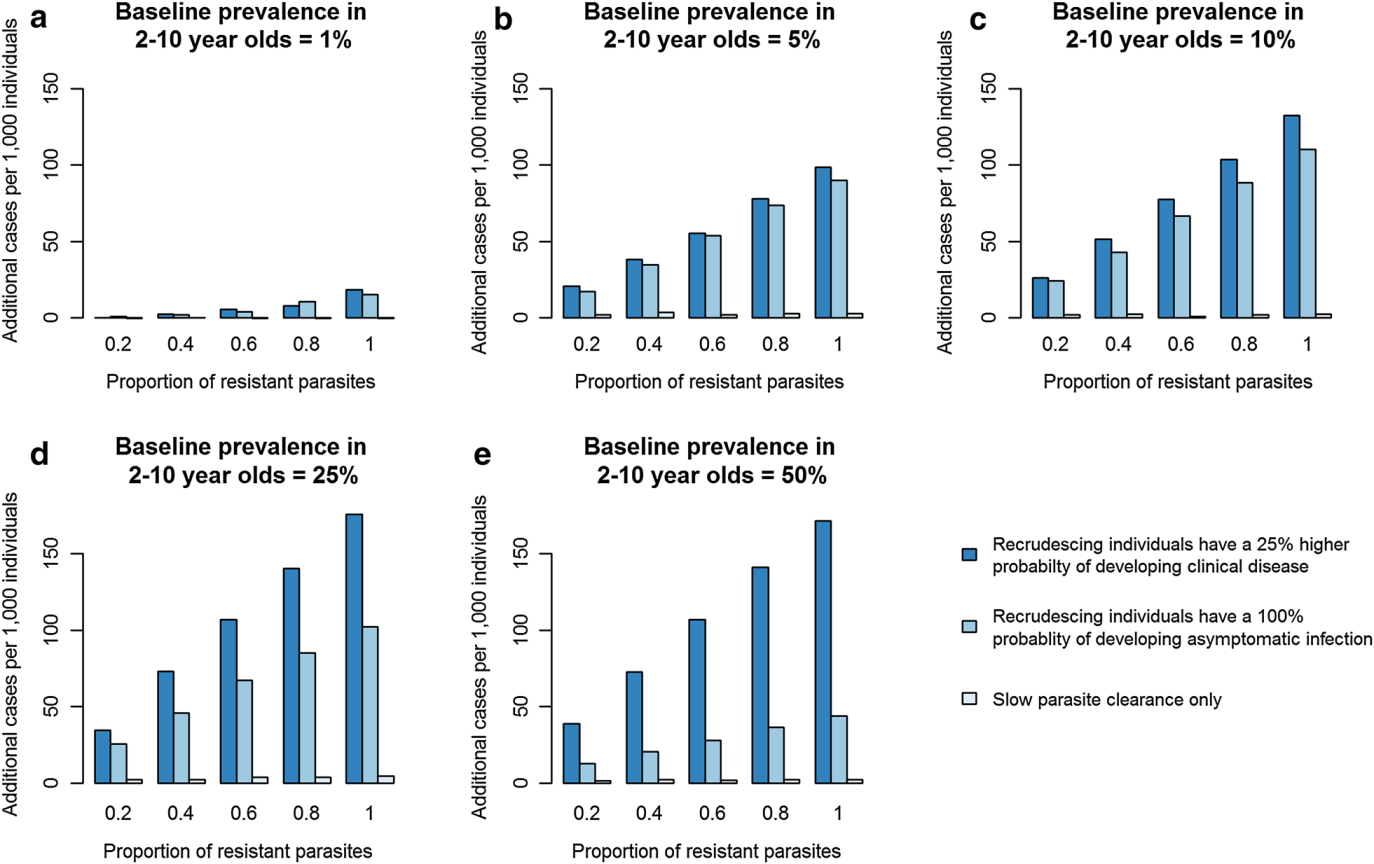
Impact of three treatment outcomes of ACT resistance on clinical incidence (mean number of cases of malaria per 1000 individuals per year) in five different transmission settings (baseline parasite prevalence in 2–10 year olds = 1, 5, 10, 25 and 50 %). The *dark blue bars* (LCF) show the situation where recrudescing individuals have a 25 % greater probability of developing clinical infection after recrudescing compared to after a new infection. The *medium blue bars* (LPF) show the situation where all recrudescing individuals develop late parasitological failure (asymptomatic patent infection). The *light blue bars* show the situation where individuals clear parasites slowly after treatment but do not go on to recrudesce. The proportion of infections recrudescing or with slow parasite clearance is shown on the *x-axis*

Figure 2, shows the estimated impact of scenarios where 20 %. 40, 60, 80 or 100 % of infections have one of three treatment outcomes: (1) LCF: individuals infected with resistant parasites always recrudesce and have a 25 % higher probability of recrudescing to clinical disease compared to infection with a sensitive parasite (dark blue bars), (2) LPF: all individuals infected with resistant parasites recrudesce to asymptomatic infection only, without clinical symptoms (medium blue bars), and 3) SPC: individuals with resistant parasites take 50 % longer to clear their infection (light blue bars).

Increasing the proportion of infections with resistant parasites that result in SPC after treatment has a small effect on clinical incidence across all transmission settings. In contrast, by increasing the proportion of infections with resistant parasites that result in recrudescence (either LCF or LPF) a substantial increase in clinical incidence is predicted. This is because LCF/LPF increases the days an individual remains infected by up to a few months whereas SPC only increases this by a few days. In high transmission settings, as expected the increase in clinical incidence is much greater when a higher proportion of recrudescing individuals develop clinical disease (LCF) rather than asymptomatic infection (LPF). However, in lower transmission settings, the relative increase in incidence resulting from LCF is only slightly greater than for LPF.

### Africa model simulations

**Fig. 3:**
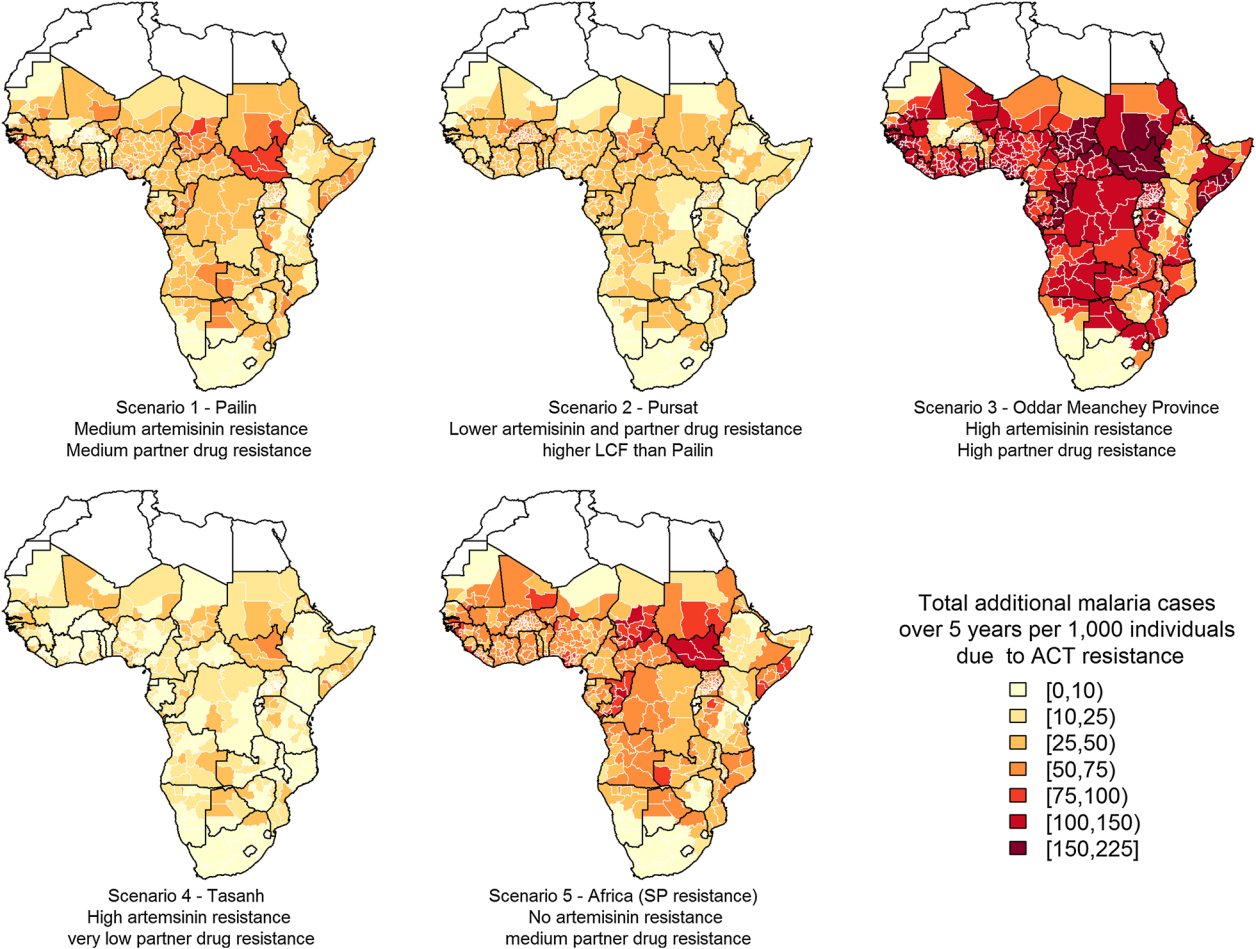
Absolute increase in clinical malaria incidence per 1000 individuals over 5 years (2016–2020) using five ACT resistance scenarios compared to a scenario with no artemisinin or partner drug resistance. All maps were created using the maptools package [30] in R

Figure 3 shows geographically-stratified estimates of the total increase in all-age clinical incidence per 1000 individuals over a 5 year period between 2016 and 2020 compared to a scenario with no artemisinin or partner drug resistance over the same time period. The increase in clinical incidence due to resistance is predicted to be highest where both artemisinin and partner drug resistance is high (Scenario 3). The scenario with high partner drug resistance and no artemisinin resistance (low SPC but higher recrudescence) (Scenario 5) has a considerably worse outcome that the scenario with high artemisinin resistance (slow parasite clearance and low recrudescence) (Scenario 4). This is because individuals with recrudescent infections have a much longer infectious period than those with SPC and lead to increased transmission. Also, these individuals can become re-infected with partner drug resistant parasites during the period of prophylaxis that follows treatment with an ACT. Although Scenario 2 has lower artemisinin and partner drug resistance than Scenario 1, the predicted increase in the number of cases is almost as high. This is because the probability of developing LCF rather than LPF is much higher in Scenario 2 than in Scenario 1. This reflects the result shown in Fig. 2 that the impact of resistance that gives rise to recrudescence is sensitive to the clinical outcome (symptomatic or asymptomatic) of a recrudescing individual, especially in a high transmission setting.

Table 2 shows the predicted total number of additional clinical malaria cases in each country for each resistance scenario. The baseline annual incidence per year in the absence of resistance is estimated to be approximately 249 million cases. Scenario 3, which has the highest artemisinin and partner drug resistance, results in the most severe outcome, with an estimated 78 million additional cases of malaria over the 5 year period, an increase of 7 % compared to the same 5 year period with no resistance. The scenario with high partner drug resistance only (Scenario 5) results in the next worse outcome, with an estimated additional 39 million cases.

**Table 2:** Malaria cases in 2010, total number of clinical cases of malaria over a 5 year period (2016–2020) for a scenario with no ACT resistance, and the additional malaria cases for each resistance scenario over the same time period (all in millions)

	Malaria incidence in 2010 (millions)	Total malaria cases with no ACT resistance (2016–2020) (millions)	Additional malaria cases due to ACT resistance (2016–2020) (millions)				
			Scenario 1	Scenario 2	Scenario 3	Scenario 4	Scenario 5
Angola	7.94	43.76	0.77	0.52	2.19	0.38	1.09
Benin	4.19	28.62	0.26	0.34	0.84	0.00	0.50
Botswana	0.10	0.68	0.05	0.04	0.13	0.02	0.06
Burkina Faso	9.89	54.99	0.34	0.84	0.63	0.00	0.97
Burundi	1.63	5.10	0.22	0.09	0.64	0.13	0.28
Cameroon	10.51	58.64	0.70	0.67	2.03	0.21	1.12
Central Afr Rep	2.37	12.80	0.18	0.17	0.55	0.06	0.29
Chad	5.53	28.49	0.72	0.51	2.02	0.31	1.01
Congo	1.97	10.22	0.24	0.16	0.67	0.10	0.33
Cote d’Ivoire	10.85	57.80	0.65	0.70	2.04	0.18	1.11
Djibouti	0.03	0.13	0.02	0.01	0.05	0.00	0.03
Dem Rep Congo	19.13	128.40	2.34	1.51	7.34	1.06	3.10
Eq. Guinea	0.31	1.97	0.02	0.02	0.07	0.00	0.04
Eritrea	0.35	2.76	0.20	0.07	0.61	0.11	0.28
Ethiopia	2.60	41.38	1.02	0.57	3.25	0.22	1.45
Gabon	0.58	4.64	0.04	0.06	0.16	0.01	0.09
Gambia	0.35	1.85	0.06	0.04	0.18	0.04	0.09
Ghana	8.74	59.20	0.94	0.82	2.90	0.31	1.48
Guinea	5.16	27.27	0.49	0.30	1.48	0.25	0.74
Guinea-Bissau	0.29	2.10	0.07	0.05	0.20	0.04	0.10
Kenya	3.47	23.62	0.53	0.15	2.06	0.12	0.81
Lesotho	0.00	0.00	0.00	0.00	0.00	0.00	0.00
Liberia	1.51	9.59	0.18	0.13	0.54	0.08	0.26
Malawi	6.95	36.22	0.37	0.31	1.14	0.14	0.57
Mali	6.33	48.80	0.32	0.57	0.63	0.00	0.79
Mauritania	0.59	3.12	0.11	0.07	0.33	0.07	0.17
Mozambique	10.31	61.22	0.65	0.71	1.90	0.05	1.14
Namibia	0.21	1.53	0.06	0.03	0.17	0.03	0.07
Niger	5.46	34.27	0.64	0.46	1.86	0.36	1.00
Nigeria	75.33	375.69	7.02	5.04	19.84	2.80	10.12
Rwanda	0.50	1.04	0.10	0.06	0.25	0.04	0.07
Senegal	1.47	8.10	0.23	0.13	0.74	0.07	0.27
Sierra Leone	2.36	9.72	0.21	0.14	0.62	0.09	0.27
Somalia	1.06	5.70	0.42	0.16	1.27	0.20	0.53
South Africa	1.36	4.84	0.51	0.30	1.64	0.20	0.70
South Sudan	3.20	16.69	0.77	0.41	2.13	0.38	1.07
Sudan	6.68	32.64	2.01	0.77	4.90	0.95	2.29
Swaziland	0.01	0.00	0.00	0.00	0.00	0.00	0.00
Tanzania	10.45	41.10	1.22	0.82	3.45	0.56	1.69
Togo	2.82	20.03	0.21	0.27	0.62	0.01	0.36
Uganda	13.26	81.38	1.29	1.04	3.99	0.47	1.98
Zambia	2.51	28.33	0.49	0.25	1.33	0.18	0.63
Zimbabwe	0.99	5.96	0.14	0.05	0.37	0.02	0.13
Totals	249.33	1420.39	26.81	19.30	77.77	10.23	39.04

**Fig. 4:**
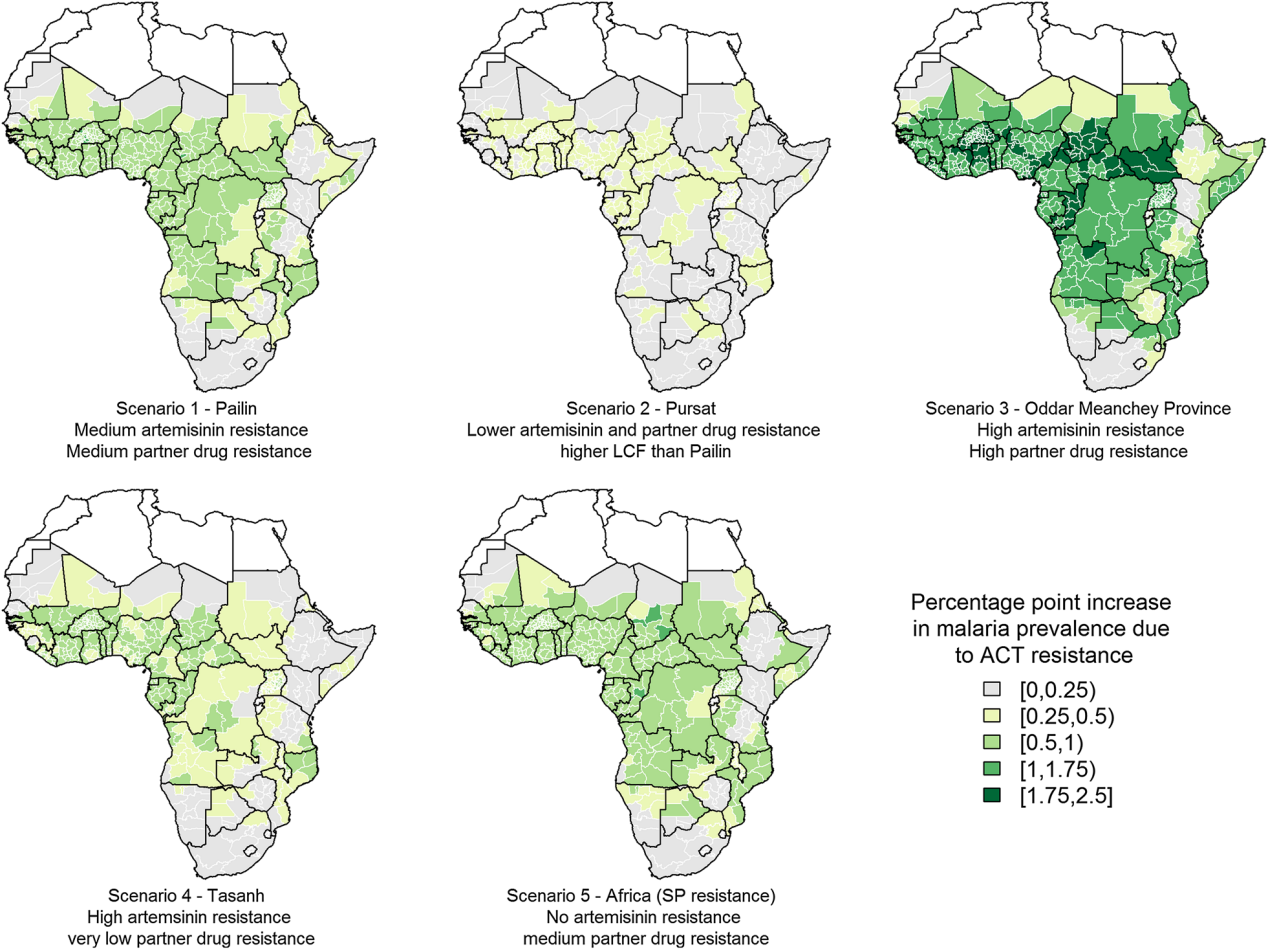
Absolute increase in malaria prevalence resulting from each ACT resistance scenario—comparing prevalence between 2016 and 2020 for scenarios with and without resistance

The absolute increase in malaria prevalence is predicted to be small for all resistance scenarios (less than 2.5 percentage points) (Fig. 4) but shows a different pattern to the increase in clinical incidence. Whilst Scenario 1 was predicted to result in only 8 million more malaria cases than Scenario 2, the predicted increase in prevalence is much greater in Scenario 1. This is because in Scenario 2 a higher proportion of individuals that recrudesce are assumed to develop clinical disease and are treated, so resistance has less impact on parasite carriage. The highest increase in prevalence is estimated for Scenario 3 with high artemisinin and partner drug resistance.

## Discussion

Based on the current levels of artemisinin and partner drug resistance observed in Asia and Africa, our results suggest that partner drug resistance is likely to result in a greater increase in transmission and incidence of uncomplicated malaria than artemisinin resistance alone. Overall, an additional 39 million cases of malaria are estimated to occur over a 5 year period in Africa if partner drug resistance levels were similar everywhere to those observed for SP resistance in parts of East Africa, relative to a scenario with no resistance (a 2.7 % increase in overall incidence of uncomplicated malaria). In Cambodia, where current parasite prevalence is very low (~1 % [11]), the model suggests an increase in the proportion of individuals recrudescing will only result in a small increase in clinical incidence. In contrast, in African settings with higher parasite prevalence, partner drug resistance is predicted to have a greater impact on morbidity, particularly if the proportion of recrudescing individuals developing LCF is high. If ACT resistance in Africa was similar to the highest levels of artemisinin and partner drug resistance currently observed in Oddar Meanchey in Cambodia, it is estimated that there would be 78 million additional clinical malaria cases between 2016 and 2020.

This study highlights the importance of protecting the effectiveness of partner drugs in Africa for two reasons. First, partner drug resistance in itself could result in higher clinical incidence than artemisinin resistance alone. Second, a failing partner drug means that parasites surviving the artemisinin phase of ACT treatment could be transmitted onwards, potentially increasing the prevalence and spread of artemisinin resistance. This has implications for investment in drug development, as it is important to maintain a pipeline of back-up partner drugs as well as investing in potential alternatives for the artemisinin derivatives. It also has implications for the surveillance of resistance, with monitoring of treatment outcomes and molecular markers of partner drug resistant parasites in sentinel sites equally as important as artemisinin resistance markers.

Although in some of the scenarios considered, partner drug resistance has more impact than low levels of artemisinin resistance, in such situations it may be possible to switch to an alternative and halt or reverse the spread of resistance (as has been observed in Tanzania with chloroquine [28]) whereas there are currently no available alternatives to artemisinin. This means that if artemisinin resistance does appear in Africa, constraining it could potentially be much more challenging than addressing the issue of partner drug resistance. Alongside the potential development of resistance to artemisinin combination therapies in Africa, there are several other threats to the significant progress made in reducing malaria transmission. These include the spread of resistance to insecticides used to treat bed nets and for indoor residual spraying, the increased ecological suitability of areas to support vector populations with climate change, the re-introduction of parasites to areas that have achieved local elimination, or the waning of financial support from major public health organizations.

The impact of partner drug resistance on clinical incidence is sensitive to the parameter determining the proportion of recrudescing individuals that develop clinical infection versus asymptomatic infection. Studies in Cambodia have shown that this parameter can vary substantially, with between 20 and 100 % of recrudescing individuals developing clinical symptoms [16]. However, all the Cambodia studies were conducted in areas of low prevalence (typically ≤1 % [11]) where acquired immunity is also likely to be low. In contrast, prevalence is currently considerably higher in many parts of Africa, meaning individuals are more likely to develop asymptomatic infection. To understand the potential impact of ACT resistance in Africa it is important to better understand how higher levels of acquired immunity influence the probability of whether recrudescence results in LCF versus LPF.

The model is parameterised for each first subnational administrative unit in Africa to match prevalence, seasonality patterns and treatment and LLIN coverage, however, the variation within each spatial unit is not captured in this study. Furthermore some areas have a greater quantity of data on these variables than others and, therefore, in some places there is greater uncertainty [23–26]. In this study, fixed static resistance scenarios are considered, whereas in reality the development of resistance is a dynamic process and is likely to change rapidly. Accurately capturing the development and spread of resistance would require models of resistant and sensitive parasite strains and a far greater understanding of how these strains interact and the relationship between resistant strains and worsening treatment outcomes. Further data would be needed to understand how resistance to one component of the ACT increases resistance pressures on the other and whether ACT resistance is less likely to occur in Africa where there are considerably more diverse parasite populations than in Cambodia.

In summary, the results suggest that, given the current data on artemisinin and partner drug resistance from South East Asia, treatment failure occurring due to resistance to the partner drug is likely to result in substantially worse outcomes in an African context compared with low levels of artemisinin resistance evidenced only by slow parasite clearance rates and low treatment failure rates. However, as observed in Cambodia, resistance to both components of the ACT may develop side-by-side, resulting in the worse outcome than resistance to either component alone. Slow parasite clearance alone could still result in substantial increases in morbidity and its associated mortality in an African context. Close monitoring of clinical and parasitological outcomes in sentinel sites in Africa, therefore, remains a high priority [29].

## Additional file

10.1186/s12936-015-1075-7 Supplementary methods.
