# Supplementary material for: Assessing the potential impact of artemisinin and partner drug resistance in sub-Saharan Africa
Source: Malar J. 2016 Jan 6;15:10. doi: 10.1186/s12936-015-1075-7 (PMC4704433; doi:10.1186/s12936-015-1075-7)
Supplement: Supplementary file 1 — 10.1186/s12936-015-1075-7 Supplementary methods. [file 12936_2015_1075_MOESM1_ESM.docx]

**Supplementary Methods**

**Assessing the potential impact of artemisinin and partner drug resistance in Sub-Saharan Africa**

Hannah C. Slater PhD ^a^, Jamie T. Griffin PhD ^a^, Azra C. Ghani PhD ^a^, Lucy C. Okell PhD ^a^

a) MRC Centre for Outbreak Analysis & Modelling, Department of Infectious Disease Epidemiology, Imperial College London, UK, W2 1PG

**Malaria transmission model with artemisinin and partner drug resistance**

A previously described model^1^ was extended to include the outcomes associated with artemisinin and partner drug resistance. This consisted of:

1. Slow parasite clearance
2. Late clinical failure
3. Late parasitological failure
4. Early treatment failure
5. Re-infection with resistant parasites whilst prophylactically protected

The new model structure is shown in Figure S1 and the original model equations are given below. We then provide a step-by-step description of how the artemisinin and partner drug resistant outcomes were included.


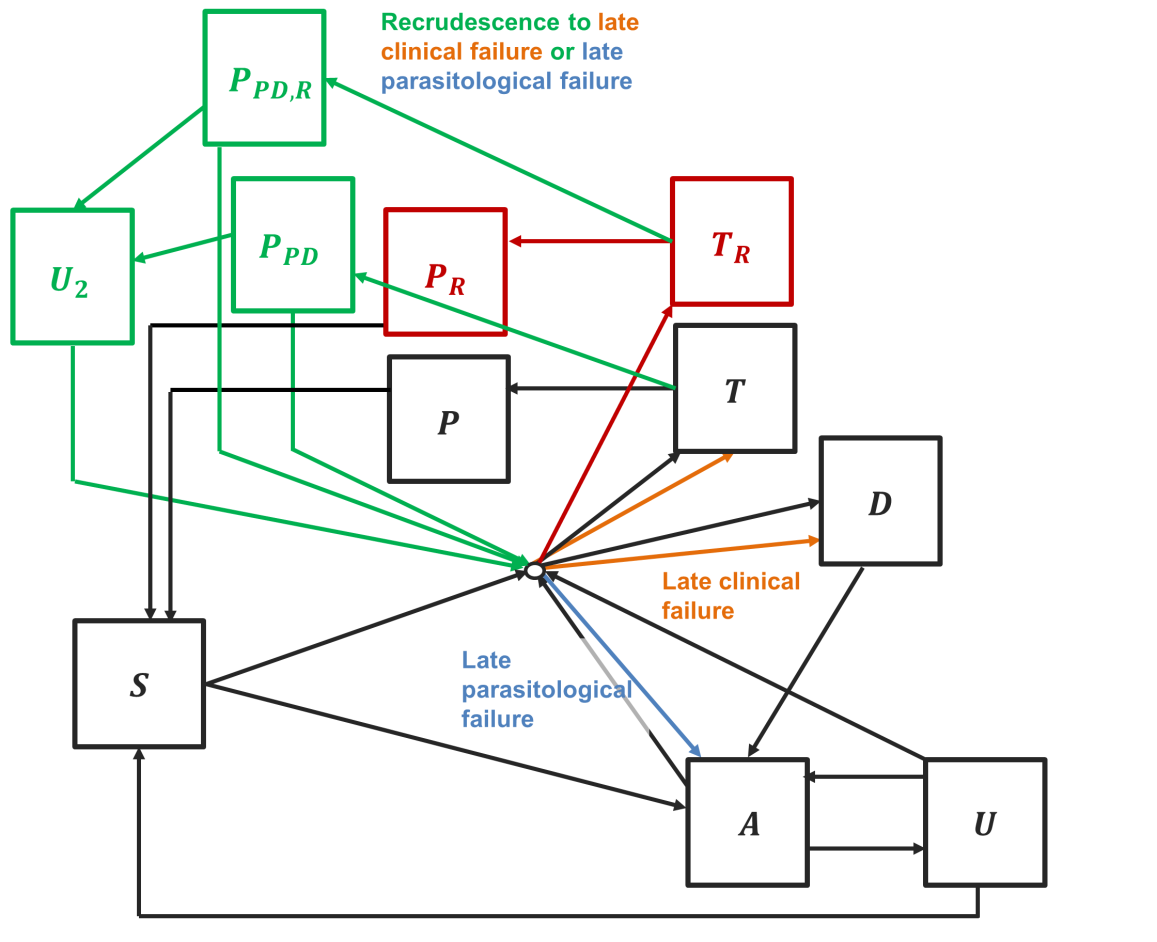


Figure S1: Model structure. The black compartments represent the original model. The red compartment and arrows represent the impact of slow parasite clearance on transmission, the green compartment and arrows show the how recrudescence affects transmission, and the blue and orange arrows demonstrate how recrudescing individuals can develop late parasitological failure or late clinical failure respectively.

**Original model equations**

$$\frac{\partial S}{\partial t}+\frac{\partial S}{\partial a}= -\Lambda S+P/d_{P}+U/d_{U}$$

$$\frac{\partial T}{\partial t}+\frac{\partial T}{\partial a}=\phi f_{T}\Lambda\left( S+A+U \right)-T/d_{T}$$

$$\frac{\partial D}{\partial t}+\frac{\partial D}{\partial a}=\phi\left( 1-f_{T} \right)\Lambda\left( S+A+U \right)-D/d_{D}$$

$$\frac{\partial A}{\partial t}+\frac{\partial A}{\partial a}=\left( 1-\phi\right)\Lambda\left( S+U \right)+D/d_{P}-\phi\Lambda A-A/d_{A}$$

$$\frac{\partial U}{\partial t}+\frac{\partial U}{\partial a}=A/d_{A}-U/d_{U}-\Lambda U$$

$$\frac{\partial P}{\partial t}+\frac{\partial P}{\partial a}=T/d_{T}-P/d_{P}$$

An individual can be in one of the six states: susceptible (S), treated clinical disease (T), untreated clinical disease (D), patent asymptomatic infection (A), sub-patent asymptomatic infection (U) or prophylactically protected following treatment (P). A full description of model parameters is provided in tables S1 and S2.

We assume that the proportion of parasites that are resistant is fixed in a given scenario, and denoted $p_{RES}$. This value represents the probability that a new infection is resistant to either artemisinin or the partner drug.

**1. Slow parasite clearance**

The proportion of individuals with artemisinin resistant parasites in each scenario is defined as the proportion of individuals with parasites on day 3 after treatment. A proportion $\left( p_{RES} \right)$ of new infections are with resistant parasites, and a proportion $\left( p_{A} \right)$ of these resistant infections will be artemisinin resistant. Individuals with an artemisinin resistant infection are assumed to clear parasites at a slower rate. This is modelled by including a new compartment $T_{R}$ which has a longer mean duration $d_{T2}$.

The mean duration spent in the prophylactically protected compartment by an individual that has cleared an artemisinin resistant infection $\left( P_{R} \right)$ is shorter than for an individual that has clearer an artemisinin sensitive infection $(P)$ so that the combined duration spent in the treated and protected compartments are the same for individuals with sensitive and resistant parasites (i.e $d_{T}+d_{P}=d_{T2}+d_{P2}$). Although the duration of prophylactic protection is denoted in the equations below as being exponentially distributed, in the model implementation an individual has a protection profile whereby the probability of being re-infected after treatment is lowest directly after treatment and wanes up until a point where their susceptibility is solely determined by their acquired pre-erythrocytic immunity. ^1^

Then the new equations for the treated and prophylactically protected compartments are as follow:

$\frac{\partial T}{\partial t}+\frac{\partial T}{\partial a}=\phi(1-p_{RES}p_{A}) f_{T} \Lambda(S+A+U)-T/d_{T}$ [1]
$\frac{\partial T_{R}}{\partial t}+\frac{\partial T_{R}}{\partial a}=\phi p_{RES}p_{A} f_{T} \Lambda(S+A+U)-T_{R}/d_{T2}$ [2]
$\frac{\partial P}{\partial t}+\frac{\partial P}{\partial a}=T/d_{T}-P/d_{P}$ [3]
$\frac{\partial P_{R}}{\partial t}+\frac{\partial P_{R}}{\partial a}=T_{R}/d_{T2}-P_{R}/d_{P2}$ [4]

Parasite clearance time is assumed to be the time taken to clear asexual parasites. In the model described in^1^ it is assumed that treated individuals have asexual parasites for an average of 5 days and that they have gametocytes for an additional 12 days after clearing asexual parasites. The infectivity of individuals post treatment was matched to data on the onward infectivity of individuals after treatment with an ACT based on human to mosquito transmission studies.

In this study we model slow parasite clearance by increasing the mean duration that a treated individual carries asexual parasites. This figure was not reported in the studies: instead we derive a relationship between the slope of the parasite clearance curve of artemisinin resistant infections and day three positivity using data from seven studies in South East Asia.^2-8^ Firstly we construct a linear model relating day three positivity to the parasite clearance half-life. Using this relationship, the parasite clearance half-life is predicted for each of the model scenarios based on the proportion of individuals that have parasites on day three (Figure S2).

To estimate the time at which the individual is assumed to have cleared parasites, we use the detection threshold for PCR. We assume there are on average 50,000 parasites per $\mu L$ before treatment and PCR can detect at low as 1 parasite per $\mu L$, We then define the time to clear parasites as the time at which the parasite population is less than 0.002% (1/50,000) of the original density. For each model scenario (with its estimated parasite clearance half-life value) we estimate the time at which the parasite density is below the level detectable using PCR. Finally we compare these values to the estimated time to clear parasites for a scenario with 0% day three positivity (i.e. fully sensitive parasites) and scale the duration in the treated compartment in the transmission model accordingly. For example, if the time to clear resistant parasites is double the time to clear sensitive parasites, we assume the mean duration in the treated compartment is doubled.


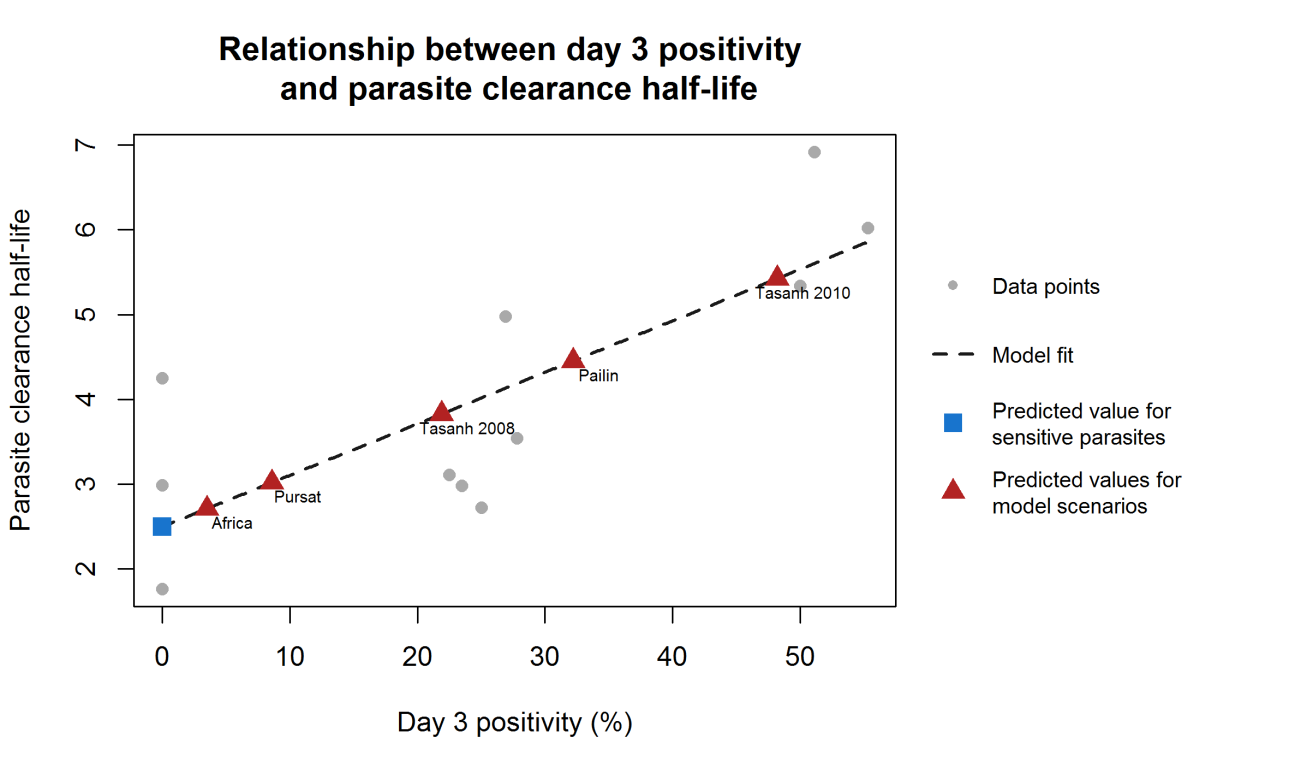


Figure S2: Relationship between day three positivity and parasite clearance half-life used to estimate parasite clearance times for the five model scenarios used in the analysis.

**2 and 3. Late clinical and late parasitological failure**

We assume that, of the infections that are resistant $(p_{RES})$, a proportion are resistant to the partner drug $\left( p_{PD} \right)$. Individuals with partner drug resistant infections initially clear parasites to sub-microscopic levels before recrudescing to either clinical (LCF) or asymptomatic infection (LPF). After initial treatment, these individuals move to a prophylactically protected with partner drug resistant parasites compartment (either $P_{PD}$ if their infection is artemisinin-sensitive or $P_{PD,R}$ if their infection is also artemisinin-resistant, as we assume individuals can have both artemisinin and partner drug resistant parasites) for a duration depending on the prophylactic properties of the drug they have taken. If the time to recrudesce is greater than the duration of the prophylactic period, then they move to a new compartment, $U_{2}$, where they have sub-microscopic infection that will recrudesce, but they are no longer prophylactically protected. Individuals in the $U_{2}$ compartment can become re-infected before recrudescing. Individuals can recrudesce from either the $P_{PD}, P_{PD,R}$ or $U_{2}$ compartments to either clinical (T/D) or asymptomatic (A) infection. Individuals with late parasitological failure recrudesce to asymptomatic infection, where they remain until their parasitaemia decreases to a sub-patent level (U) or are re-infected. The rate at which individuals with partner drug resistant parasites recrudesce is given by $r_{REC}$.

The probability of a recrudescing individual developing either clinical $(\phi)$ or asymptomatic infection $(1-\phi)$ is dependent on the level of immunity to clinical disease, determined by the previous exposure to infection.^1^ We consider the possibility that recrudescing infections have an altered probability of becoming either clinical or asymptomatic. This is modelled by having a parameter $R_{\phi}$ which determines whether a recrudescing infection has a greater or lower probability of becoming clinical rather than asymptomatic relative to a new infection. Values less than one indicate a lower probability of developing clinical infection. Values for $R_{\phi}$ were found by firstly running the original transmission model with an equilibrium prevalence of 1% for 2-10 year olds (for SE Asian scenarios) and 7% prevalence in 2-10 year olds (for the African scenario) based on local transmission in the settings considered. The proportion of new infections that are clinical was outputted for each simulation and then scaled (by $R_{\phi}$) to match the proportion of recrudescing individuals developing clinical infection in each scenario. The relevant value of $R_{\phi}$ was used in each scenario for the model simulations across Africa.

$\frac{\partial P}{\partial t}+\frac{\partial P}{\partial a}=\left( 1-p_{RES}p_{PD} \right)T/d_{T}-P/d_{P}$ [5]

$\frac{\partial P_{R}}{\partial t}+ \frac{\partial P_{R}}{\partial a}={{\left( 1-p_{RES}p_{PD} \right) T}_{R}}/{d_{T2}}-{P_{R}}/{d_{P2}}$ [6]

$\frac{\partial P_{PD}}{\partial t}+\frac{\partial P_{PD}}{\partial a}=p_{RES}p_{PD}T/d_{T}-P_{PD}/d_{P}-r_{REC}P_{PD}$ [7]

$\frac{\partial P_{PD,R}}{\partial t}+\frac{\partial P_{PD,R}}{\partial a}=p_{RES}p_{PD}T_{R}/d_{T2}-P_{PD,R}/d_{P2}-r_{REC}P_{PD,R}$ [8]

$\frac{\partial U_{2}}{\partial t}+\frac{\partial U_{2}}{\partial t}={P_{PD}}/{d_{P}} +P_{PD,R}{/d}_{P2}-r_{REC}U_{2}$ [9]

$\frac{\partial D}{\partial t}+\frac{\partial D}{\partial a}=\phi\left( 1-f_{T} \right)\Lambda(S+A+U)-D/d_{D}+\left( 1-f_{T} \right) \phi R_{\phi} r_{REC}(P_{PD}+P_{PD,R}+ U_{2})$ [10]

$\frac{\partial A}{\partial t}+\frac{\partial A}{\partial a}=\left( 1-\phi\right)\Lambda\left( S+U \right)+\frac{D}{d_{D}}-\phi\Lambda A-A/d_{A}+\left( 1-\phi R_{\phi} \right)r_{REC}(P_{PD}+P_{PD,R}+ U_{2})$ [11]

$\frac{\partial T}{\partial t}+\frac{\partial T}{\partial a}=\phi{\left( 1-p_{RES}p_{A} \right)f}_{T} \Lambda\left( S+A+U \right)-T/d_{T}+f_{T} \left( 1-p_{RES}p_{A} \right)\phi R_{\phi}r_{REC}(P_{PD}+P_{PD,R}+ U_{2})$ [12]

$\frac{\partial T_{R}}{\partial t}+\frac{\partial T_{R}}{\partial a}=\phi{p_{RES}p_{A} f}_{T} \Lambda(S+A+U)-T_{R}/d_{T2}+{p_{RES}p_{A} f}_{T} \phi R_{\phi}r_{REC}(P_{DP}+P_{PD,R}+ U_{2})$ [13]

**4. Early treatment failure**

Although not considered in this study, we included this treatment outcome in the model. We assume that early treatment failure results in the ACT failing to halt the onset of clinical disease. This is modelled by assuming that for a proportion $p_{ETF}$ of treated resistant infections, the treatment has no effect. Equations [10], [12] and [13] now become:

$\frac{\partial D}{\partial t}+\frac{\partial D}{\partial a}=\phi\left( 1-f_{T}+f_{T}p_{ETF} \right)\Lambda(S+A+U)-D/d_{D}+\left( 1-f_{T}+f_{T}p_{ETF} \right) \phi R_{\phi} r_{REC}(P_{PD}+P_{PD,R}+ U_{2})$ [14]

$\frac{\partial T}{\partial t}+\frac{\partial T}{\partial a}=\phi{\left( 1-p_{RES}p_{A} \right)\left( 1-p_{ETF} \right) f}_{T} \Lambda\left( S+A+U \right)-T/d_{T}+f_{T} \left( 1-p_{ETF} \right) \left( 1-p_{RES}p_{A} \right)\phi R_{\phi}r_{REC}(P_{PD}+P_{PD,R}+ U_{2})$ [15]

$\frac{\partial T_{R}}{\partial t}+\frac{\partial T_{R}}{\partial a}=\phi{p_{RES}p_{A} \left( 1-p_{ETF} \right) f}_{T} \Lambda(S+A+U)-T_{R}/d_{T2}+{p_{RES}p_{A} f}_{T} \left( 1-p_{ETF} \right) \phi R_{\phi}r_{REC}(P_{DP}+P_{PD,R}+ U_{2})$ [16]

**5. Re-infection with resistant parasites whilst prophylactically protected**

We assume that prophylactically protected individuals can get re-infected if they are challenged with a partner-drug resistant infection. Therefore, the force of infection on protected individuals is the normal force of infection multiplied by the proportion of infections that are resistant to the partner drug. Therefore, equations [5-8] become:

$\frac{\partial P}{\partial t}+\frac{\partial P}{\partial a}={\left( 1-p_{RES}p_{PD} \right)T}/{d_{T}}-P/{d_{P}}-p_{RES} p_{PD}\Lambda P$ [17]

$\frac{\partial P_{R}}{\partial t}+\frac{\partial P_{R}}{\partial a}={\left( 1-p_{RES}p_{PD} \right)T_{R}}/{d_{T2}}-{P_{R}}/{d_{P2}}-p_{RES} p_{PD}\Lambda P{}_{R}$ [18]

$\frac{\partial P_{PD}}{\partial t}+\frac{\partial P_{PD}}{\partial a}=p_{RES}p_{PD}T/d_{T}-P_{PD}/d_{P}-r_{REC}P_{PD}-p_{RES} p_{PD}\Lambda P{}_{PD}$ [19]

$\frac{\partial P_{PD,R}}{\partial t}+\frac{\partial P_{PD,R}}{\partial a}=p_{RES}p_{PD}T_{R}/d_{T2}-P_{PD,R}/d_{P2}-r_{REC}P_{PD,R}-p_{RES} p_{PD}\Lambda P{}_{PD,R}$ [20]

Also, individuals in the $U_{2}$ compartment can get re-infected with any parasite

$\frac{\partial U_{2}}{\partial t}+\frac{\partial U_{2}}{\partial t}={P_{PD}}/{d_{P}} +P_{PD,R}{/d}_{P2}-r_{REC}U_{2}-\Lambda U_{2}$ [21]

Therefore all individuals in $P, P_{R},P_{PD},P_{PD,R}$ and $U_{2}$ that get re-infected (given by $p_{RES}p_{PD}\Lambda P, p_{RES}p_{PD}\Lambda P_{R},p_{RES}p_{PD}\Lambda P_{PD}, p_{RES}p_{PD}\Lambda P_{PD,R}$ and $\Lambda U_{2}$ respectively) will now transition to either $T,T_{R}, D$or $A$ as shown below in the complete equations for the new model.

**Final artemisinin and partner drug resistance malaria transmission model**

$\frac{\partial S}{\partial t}+\frac{\partial S}{\partial a}= -\Lambda S+\left( P/{d_{P}}+{P_{R}}/{d_{P2}} \right)+U/d_{U}$

$\frac{\partial T}{\partial t}+\frac{\partial T}{\partial a}=\phi{\left( 1-p_{RES}p_{A} \right)f}_{T} \left( 1-p_{ETF} \right) \Lambda\left( S+A+U+U_{2}+p_{RES}p_{PD}(P+P_{R}+P_{PD}+P_{PD,R}) \right) -T/{d_{T}}+f_{T}\left( 1-p_{ETF} \right) \left( 1-p_{RES}p_{A} \right)\phi R_{\phi}r_{REC}(P_{PD}+P_{PD,R}+ U_{2})$

$\frac{\partial T_{R}}{\partial t}+\frac{\partial T_{R}}{\partial a}=\phi{p_{RES}p_{A} f}_{T} \left( 1-p_{ETF} \right) \Lambda\left( S+A+U+U_{2}+p_{RES}p_{PD}(P+P_{R}+P_{PD}+P_{PD,R}) \right)-{T_{R}}/{d_{T2}}+{p_{RES}p_{A} f}_{T} \left( 1-p_{ETF} \right) \phi R_{\phi} r_{REC}(P_{PD}+P_{PD,R}+ U_{2})$

$\frac{\partial D}{\partial t}+\frac{\partial D}{\partial a}=\phi\left( 1-f_{T}+f_{T}p_{ETF} \right)\Lambda\left( S+A+U+U_{2}+p_{RES}p_{PD}(P+P_{R}+P_{PD}+P_{PD,R}) \right)-D/d_{D}+\left( 1-f_{T}+f_{T}p_{ETF} \right) \phi R_{\phi} r_{REC}(P_{PD}+P_{PD,R}+ U_{2})$

$\frac{\partial A}{\partial t}+\frac{\partial A}{\partial a}=\left( 1-\phi\right)\Lambda\left( S+U+U_{2}+p_{RES}p_{PD}(P+P_{R}+P_{PD}+P_{PD,R}) \right)+D/{d_{D}}-\phi\Lambda A-A/{d_{A}}+\left( 1-\phi R_{\phi} \right)r_{REC}(P_{PD}+P_{PD,R}+ U_{2})$

$\frac{\partial U}{\partial t}+\frac{\partial U}{\partial a}=A/d_{A}-U/d_{U}-\Lambda U$

$\frac{\partial U_{2}}{\partial t}+\frac{\partial U_{2}}{\partial t}={P_{PD}/d_{P} +P}_{PD,R}/d_{P2}-r_{REC}U_{2}-\Lambda U_{2}$

$\frac{\partial P}{\partial t}+\frac{\partial P}{\partial a}={\left( 1-p_{RES}p_{PD} \right)T}/{d_{T}}-P/{d_{P}}-p_{RES} p_{PD}\Lambda P$

$\frac{\partial P_{R}}{\partial t}+\frac{\partial P_{R}}{\partial a}={\left( 1-p_{RES}p_{PD} \right)T_{R}}/{d_{T2}}-{P_{R}}/{d_{P2}}-p_{RES} p_{PD}\Lambda P{}_{R}$

$\frac{\partial P_{PD}}{\partial t}+\frac{\partial P_{PD}}{\partial a}=p_{RES}p_{PD}T/d_{T}-P_{PD}/d_{P}-r_{REC}P_{PD}-p_{RES} p_{PD}\Lambda P{}_{PD}$

$\frac{\partial P_{PD,R}}{\partial t}+\frac{\partial P_{PD,R}}{\partial a}=p_{RES}p_{PD}T_{R}/d_{T2}-P_{PD,R}/d_{P2}-r_{REC}P_{PD,R}-p_{RES} p_{PD}\Lambda P{}_{PD,R}$

**State variables**

| **State variable** | **Description** |
| --- | --- |
| $S(t,a)$ | Susceptible individuals at time $t$ and age $a$ |
| $T(t,a)$ | Treated (artemisinin sensitive parasites) |
| $T_{R}(t,a)$ | Treated (artemisinin resistant parasites) |
| $P\left( t,a \right)$ | Prophylactically protected (artemisinin sensitive, partner drug sensitive parasites) |
| $P_{R}(t,a)$ | Prophylactically protected (artemisinin resistant, partner drug sensitive parasites) |
| $P_{PD}(t,a)$ | Prophylactically protected (artemisinin sensitive, partner drug resistant parasites) |
| $P_{PD,R}(t,a)$ | Prophylactically protected (artemisinin resistant, partner drug resistant parasites) |
| $U_{2}\left( t,a \right)$ | Sub-patent infection with partner drug resistant parasites that will recrudesce |
| $D\left( t,a \right)$ | Clinical disease |
| $A(t,a)$ | Asymptomatic patent infection |
| $U\left( t,a \right)$ | Asymptomatic sub-patent infection |

Table S1: State variables

| **Parameter** | **Description** |
| --- | --- |
| $\phi$ | Probability of acquiring clinical disease upon infection |
| $R_{\phi}$ | Relative risk of developing clinical infection after recrudescing compared to a new infection |
| $p_{RES}$ | Proportion of infections that are (artemisinin or partner drug) resistant |
| $p_{A}$ | Proportion of resistant infections that are artemisinin resistant |
| $p_{PD}$ | Proportion of resistant infections that are partner drug resistant |
| $r_{REC}$ | Rate of recrudescing with partner drug resistant parasites |
| $p_{ETF}$ | Proportion of resistant infections that result in early treatment failure |
| $d_{T}$ | Mean duration of treated clinical disease (with artemisinin sensitive infection) |
| $d_{T2}$ | Mean duration of treated clinical disease (with artemisinin resistant infection) |
| $d_{P}$ | Mean duration of prophylactic protection (with artemisinin sensitive infection) |
| $d_{P2}$ | Mean duration of prophylactic protection (with artemisinin resistant infection) |
| $d_{D}$ | Mean duration of untreated clinical disease |
| $d_{A}$ | Mean duration of asymptomatic patent infection |
| $d_{U}$ | Mean duration of asymptomatic sub-patent infection |
| $f_{T}$ | Proportion of clinical infections treated |
| $\Lambda$ | Force of infection from vectors to humans |
| $d_{E}$ | Duration of latent host infection |

Table S2: Model Parameters

**Data maps used to create the first administrative level estimates of artemisinin and partner drug resistance**

Simulations were run at resolution of the first administrative unit across Africa (sub-national regions). Five data maps were used to inform the underlying spatial heterogeneity in transmission and intervention coverage across the continent: 1) slide prevalence in 2-10 year olds (from Malaria Atlas Project)^9^, 2) the underlying population demographic data based on UN population figures^10^, 3) access to treatment coverage,^11^ (Figure S3), 4) LLIN coverage^12^, and 5) the seasonal pattern of transmission determined by high resolution rainfall data.^13,14^


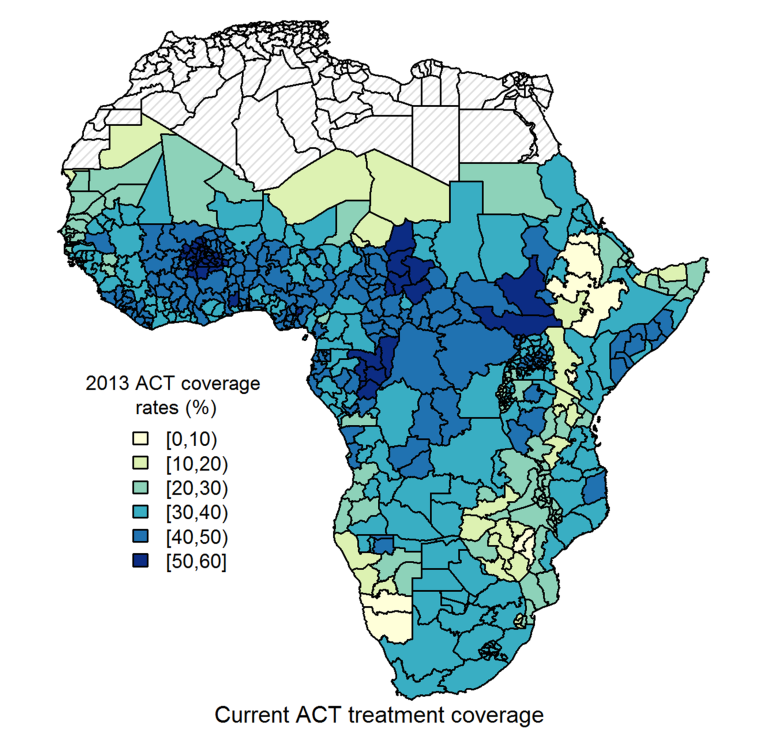


Figure S3: Proportion of clinical malaria cases that receive an ACT. The level of ACT coverage in 2010 was estimated for each first administrative unit based on the most recent household surveys and was assumed to remain constant from 2010 onwards.^11^ The map was created using the maptools^15^ package in R.

**References**

1. Griffin JT, Ferguson NM, Ghani AC. Estimates of the changing age-burden of Plasmodium falciparum malaria disease in sub-Saharan Africa. *Nat Commun* 2014; **5**.

2. Hien T, Thuy-Nhien N, Phu N, et al. In vivo susceptibility of Plasmodium falciparum to artesunate in Binh Phuoc Province, Vietnam. *Malar J* 2012; **11**(1): 1-11.

3. Starzengruber P, Swoboda P, Fuehrer H-P, et al. Current Status of Artemisinin-Resistant *falciparum* Malaria in South Asia: A Randomized Controlled Artesunate Monotherapy Trial in Bangladesh. *PLoS One* 012; **7**(12): e52236.

4. Dondorp AM, Nosten F, Yi P, et al. Artemisinin Resistance in Plasmodium falciparum Malaria. *N Engl J Med* 2009; **361**(5): 455-67.

5. Bethell D, Se Y, Lon C, et al. Artesunate Dose Escalation for the Treatment of Uncomplicated Malaria in a Region of Reported Artemisinin Resistance: A Randomized Clinical Trial. *PLoS One* 2011; **6**(5): e19283.

6. Das D, Tripura R, Phyo AP, et al. Effect of High-Dose or Split-Dose Artesunate on Parasite Clearance in Artemisinin-Resistant Falciparum Malaria. *Clin Infect Dis* 2013; **56**(5): e48-e58.

7. Kyaw MP, Nyunt MH, Chit K, et al. Reduced Susceptibility of *Plasmodium falciparum* to Artesunate in Southern Myanmar. *PLoS One* 2013; **8**(3): e57689.

8. Mayxay M, Khanthavong M, Chanthongthip O, et al. No Evidence for Spread of Plasmodium falciparum Artemisinin Resistance to Savannakhet Province, Southern Laos. *Am J Trop Med Hyg* 2012; **86**(3): 403-8.

9. Gething PW, Patil AP, Smith DL, et al. A new world malaria map: Plasmodium falciparum endemicity in 2010. *Malar J* 2011; **10**(1): 378.

10. UN. , Department of Economic & Social Affairs, Population Division. World Population Prospects, the 2010 Revision. http://esa.un.org/wpp/ Accessed Feb 2013. 2010.

11. Cohen JM, Woolsey AM, Sabot OJ, Gething PW, Tatem AJ, Moonen B. Public health. Optimizing investments in malaria treatment and diagnosis. *Science* 2012; **338**(6107): 612-4.

12. Flaxman AD, Fullman N, Otten MW, Jr., et al. Rapid scaling up of insecticide-treated bed net coverage in Africa and its relationship with development assistance for health: a systematic synthesis of supply, distribution, and household survey data. *PLoS Med* 2010; **7**(8): e1000328.

13. Cairns M, Roca-Feltrer A, Garske T, et al. Estimating the potential public health impact of seasonal malaria chemoprevention in African children. *Nat Commun* 2012; **3**: 881.

14. Griffin JT, Hollingsworth TD, Okell LC, et al. Reducing Plasmodium falciparum malaria transmission in Africa: a model-based evaluation of intervention strategies. *PLoS Med* 2010; **7**(8): e1000324.

15. Roger Bivand and Nicholas Lewin-Koh (2014). maptools: Tools for reading and handling

spatial objects. R package version 0.8-29. http://CRAN.R-project.org/package=maptools.
